# Dietary exposure assessments for children in europe (the EXPOCHI project): rationale, methods and design

**DOI:** 10.1186/0778-7367-69-4

**Published:** 2011-10-24

**Authors:** Inge Huybrechts, Isabelle Sioen, Polly E Boon, Jiri Ruprich, Lionel Lafay, Aida Turrini, Pilar Amiano, Tero Hirvonen, Melissa De Neve, Davide Arcella, Joanna Moschandreas, Anna Westerlund, Lourdes Ribas-Barba, Annett Hilbig, Stalo Papoutsou, Tue Christensen, Maciej Oltarzewski, Suvi Virtanen, Irena Rehurkova, Mikel Azpiri, Stefania Sette, Mathilde Kersting, Alicja Walkiewicz, Luis Serra-Majem, Jean-Luc Volatier, Ellen Trolle, Michael Tornaritis, Leif Busk, Anthony Kafatos, Stefan Fabiansson, Stefaan De Henauw, Jacob D Van Klaveren

**Affiliations:** 1 Department of Public Health, Ghent University, University Hospital, Ghent, Belgium; 2 RIKILT - Institute of Food Safety, Wageningen University and Research centre, Wageningen, The Netherlands; 3 Department of Food Safety and Nutrition, National Institute of Public Health, Czech Republic; 4 Agence Française de Sécurité Sanitaire des Aliments (AFFSA), France; 5 National Institute for Research on Food and Nutrition (INRAN), Rome, Italy; 6 Public Health Division of Gipuzkoa, Basque Covernement, San Sebastian, CIBER Epidemiología y Salud Pública (CIBERESP), Spain; 7 Risk Assessment Unit, Finnish Food Safety Authority, Helsinki, Finland; 8 European Food Safety Authority, Parma, Italy; 9 Department of Social Medicine, Faculty of Medicine, University of Crete, Greece; 10 Livsmedelsverket, NFA - National Food Administration, Sweden; 11 Nutrition Research Foundation, Science Park of the University of Barcelona, Spain; 12 Research Institute of Child Nutrition, Rheinische Friedrich-Wilhelms-Universitaet Bonn, Germany; 13 Research and Education Institute of Child Health, Strovolos, Cyprus; 14 Department of Nutrition, National Food Institute, Technical University of Denmark, Søborg, Denmark; 15 National Food and Nutrition Institute, Warsaw, Poland; 16 Nutrition Unit, National Institute for Health and Welfare, Helsinki, Finland; 17 Department of Clinical Sciences, University of Las Palmas de Gran Canaria, Spain

**Keywords:** Food, dietary exposure assessment, children, Europe, design, concentration data, health risk, consumption data, lead, chromium, selenium, food colours

## Abstract

**Background/purpose:**

The number of dietary exposure assessment studies focussing on children is very limited. Children are however a vulnerable group due to their higher food consumption level per kg body weight. Therefore, the EXPOCHI project aims [1] to create a relational network of individual food consumption databases in children, covering different geographical areas within Europe, and [2] to use these data to assess the usual intake of lead, chromium, selenium and food colours.

**Methods:**

EXPOCHI includes 14 food consumption databases focussed on children (1-14 y old). The data are considered representative at national/regional level: 14 regions covering 13 countries. Since the aim of the study is to perform long-term exposure assessments, only data derived from 24 hr dietary recalls and dietary records recorded on at least two non-consecutive days per individual were included in the dietary exposure assessments. To link consumption data and concentration data of lead, chromium and selenium in a standardised way, categorisation of the food consumption data was based on the food categorisation system described within the SCOOP Task report 3.2.11. For food colours, the food categorisation system specified in the Council Directive 94/36/EC was used.

**Conclusion:**

The EXPOCHI project includes a pan-European long-term exposure assessment of lead, chromium, selenium and food colours among children living in 13 different EU countries. However, the different study methods and designs used to collect the data in the different countries necessitate an in-depth description of these different methods and a discussion about the resulting limitations.

## Introduction

Until now, the number of dietary exposure assessment studies focussing on children has been very limited [1-4]. Most studies that have been published focused on dietary/chemical intakes among adults. It has, however, been recognised that children may be a potentially vulnerable subgroup in this respect. Children consume more food and water compared to adults when expressed per kg body weight, resulting in relatively higher exposures to adverse compounds [5]. Also specific dietary patterns of children may contribute to a higher exposure to contaminants present in food. Apart from having a higher exposure, children also have a different physiology from that of adults. Due to the development of different organ systems during childhood, children may be more sensitive to neurotoxic, endocrine, and immunological toxic effects up to 4 years of age. This is also true for children aged 5 up to 12 years, although to a lesser extent for immunological toxic effects [6]. Due to these differences between adults and children regarding exposure and physiology, it is important to address children as a separate subgroup in risk assessments.

Therefore, the project described in this paper aimed to provide important input data to estimate the dietary exposure to selected additives and chemical substances present in foods among children. This project, called 'Individual food consumption data and exposure assessment studies for children' (acronym: EXPOCHI), was financed by the European Food Safety Authority (EFSA) and ran for 13 months, from 1 November 2008 until 30 November 2009.

The objective of the EXPOCHI project was to create a relational network of different individual food consumption databases in children, representative of diverse regions/countries within Europe, covering different geographical areas and to use these data for four dietary exposure assessment studies. The individual food consumption data present in this network were harmonised and made available to EFSA for future exposure assessments to be performed by EFSA in the frame of EFSA opinions.

In this paper we describe the rationale behind the EXPOCHI project and the methods, procedures and study design used to reach its objectives. An in-depth description of, and discussion about, the standardised food categorisation systems used within this project will be provided in a later publication. Also further details about the four specific exposure assessments and their results will be provided in later publications regarding the exposure to the individual compounds lead, chromium, selenium and food colours. The main objective of this paper is to describe and discuss the study design and methods used within the EXPOCHI project so that future manuscripts describing the results of the four exposure assessments, and possible future exposure assessments executed in the frame of the EFSA opinions, can refer to this paper for the in-depth project description.

## Methods and Design

### 1. Compilation of individual food consumption data among children in Europe

**Figure 1:**
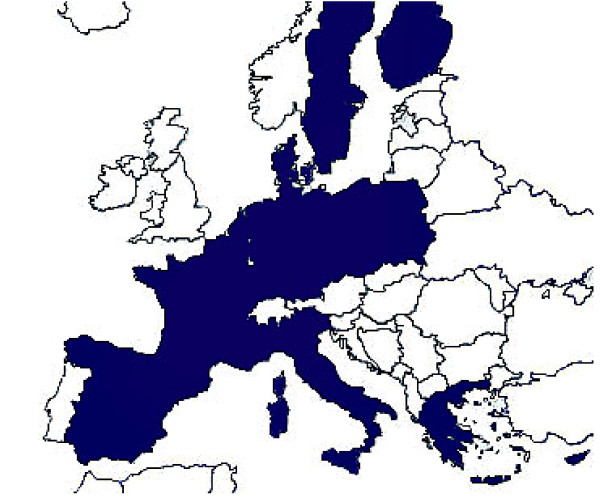
**Countries included in the EXPOCHI project (coloured in black): Belgium, Cyprus, Czech Republic, Denmark, Finland, France, Germany, Greece, Italy, Poland, Spain, Sweden & The Netherlands**.

As mentioned above, this project aimed at structuring in a harmonised way different individual food consumption databases on children (1-14 years old), representative of diverse regions/countries within Europe, and covering different geographical areas, as a basis for exposure assessments carried out for/by EFSA. The EXPOCHI consortium consisted of 14 different European partners, representing 13 different European countries (Figure 1), each providing a database with individual food consumption data (two different regions of Spain were involved providing two different databases) for children 1-14 years of both genders (Table 1).

**Table 1:** Summary table describing the national/regional nutrition surveys included in the EXPOCHI project

Country	Time span fieldwork	Population	Sampling	Sampling frame	Nr. of subject	Age range (y)	Sex	PR*
Belgium	Oct 2002 - Feb 2003	Preschoolers	Multi-stage cluster design (43 schools PSU randomly selected in the five different provinces of Flanders)	Register of schools from Ministry of Education	696 (3 days) 1027 (> 1 day)	2.5-6.5	M/F	50%

Netherlands	2006-2007	Toddlers/Preschoolers	Random sampling stratified by age and gender	Consumer panels	1279	2-6	M/F	78%

France	Dec 2005 - May 2007	All age and gender groups	Multi-stage cluster design	National census for 1998 & additional data of houses & buildings built between 1999 & 2004	574	3-10	M/F	69%

Italy	Oct 2005 - Dec 2006	All age and gender groups	Multi-stage stratified design (households as clusters)	Telephone directories at municipality level	1329 households	1-10 (no max. age limit)	M/F	33%

Spain1	May 1998 - April 2000	All age and gender groups	Multi-stage stratified design (geographical area, population size) and randomised by clusters in two stages (municipalities, individuals).	From national census	3534 (1 day) 850 (2 days)	1-24	M/F	68%

Spain2	March 2004 - Feb 2005	children and adolescents	Multi-stage sampling design	A Regional Register from the Basque Country Statistics Institute, EUSTAT	1178 households	4-18	M/F	77%

Denmark	June 2000 - December 2002	children	Random sampling	From civil registration system	825	4-10	M/F	71.4%

Czech Republic	Nov 2003-Nov 2004	Age groups 4 - 90 years - national food consumption study	Multistage sampling design	Czech national census data from 2001	413	4-14	M/F	54%

Finland I	Ongoing cohort study	Finnish Type I Diabetes Predictin and Prevention Study	Cohort recruited based on genetic susceptibility on diabetes	All newborn babies in the area of three university hospitals	500 (altogether 1500)	1, 3, 6	M/F	NA

Finland II	Ongoing cohort study (participants were 10 yrs in 2000-2002)	Special Turku Coronary Risk Factor Intervention Study (control group)	Cohort recruited from the Turku well-baby clinics	All children aged 5 months and living in the Turku city were invited to the well-baby clinics.	278 (altogether)	> 6 years	M/F	NA

Greece	February 2004-June 2005	Preschoolers	The sample consisted of all state-run nursery schools/playgroups in one of the four prefectures of Crete	Register of schools from Ministry of Education	847 (total sample size 1755)	4 to 6	M/F	48%

Sweden	May/June - Oct/Nov 2003	Children	Multi-stage cluster design	National register (for 4 years old) and list of schools (for 8-9 and 11-12 years old) were used	2600	3 to 13	M/F	73%

Cyprus	Oct 2002 - June 2006	Children, adolescents	Multi-stage cluster design	Register of schools from Ministry of Education	1000 (3 days) 500 (1 day)	2-18 (2-10 only 1 day)	M/F	52%

Poland	Sept.-Dec. 2000	All age and gender groups from 1362 surveyed households from all area of Poland	Random sampling using Central Statistical Office internal procedure	Random subsample of Polish population	892 (from 4134)	1-14	M/F	95%

Germany	Ongoing cohort (since 1985)	German children and adolescents	An (open) cohort sample with about 40 new infants starting annually	Invitations and recruitment are mainly by study families who inform other parents about the study	> 600	1-10	M/F	NA

*Participation Rate (PR)

NA = Not applicable (cohort study)

Due to the absence of a standardised approach for the collection of food consumption data in children, this project included 14 individual food consumption databases based on different methodologies. An important condition for inclusion was that the data had to be representative at a national or a large regional level and were derived from the most recent survey (less than 10 years old at the onset of the EXPOCHI project). The oldest cross-sectional surveys dated from 1998-2000. For Germany, a cohort study (the DONALD study), which started in 1985, was included in the EXPOCHI project (the three most recent data collections, namely 2006, 2007 and 2008 were included in this EXPOCHI project). A summary table of the food consumption surveys behind the databases is provided in Table 1. This table contains an overview of the methodology used for each survey, the year(s) the data were collected, the sampling procedure and the number of subjects per age group [7-22]. When comparing the different dietary intake assessment methods used, it could be concluded that most countries used a multiple day dietary record method (diary), ranging from 2 days in The Netherlands to up to 7 days in Denmark. Three countries used the 24 hr dietary recall method to assess the dietary intake among children (Spain (2 days), Cyprus (2 days) and Poland (1 day)). All studies used parents (and/or caregivers) as a proxy for their children and both genders were represented in all surveys. Not all the food consumption databases covered all seasons of the year (Belgium: only autumn & winter, Greece: only spring and summer, Sweden: only spring and autumn). However, all surveys covered all days of the week.

In total nine surveys were conducted at national level (Cyprus, Czech Republic, Denmark, Italy, France, The Netherlands, Spain (enKid), Sweden, and Poland) and five at regional level (Belgium, Finland, Germany, Greece, Spain-Basque). Furthermore, the age ranges covered in the surveys differed greatly between surveys. Five food surveys provided food consumption data of children from age 1 onwards: Finland-DIPP (1, 3 and 6 y), Germany (1-10 y), Italy (1-10 y), Spain-enKid (1-14 y) and Poland (1-14 y). Two surveys provided data from the age of 2 onwards (Belgium and The Netherlands (2-6 y)), two from the age of 3 (France (3-10) and Sweden (3-13)), four from the age of 4 (Czech Republic (4-14 y), Denmark (4-10 y), Greece (4-6 y) and Spain-Basque (4-14 y)), and finally one from the age of 7 (Finland-STRIP (7 and 8 y)) and one from the age of 11 (Cyprus (11-14 y)).

Most surveys used a multi-stage (cluster) sampling design (Belgium, France, Italy, Spain-Basque, Spain-enKid, Cyprus and Sweden). Germany and Finland used a cohort design and in the Netherlands and Greece a convenience sample was used. Denmark, Czech Republic and Poland used a random sampling design.

The most common sampling frames used were national census data (France, Spain, Denmark, Czech Republic, Poland) and school registries (Belgium, Greece and Cyprus). The Netherlands used consumer panels and Italy a phone directory.

The participation rates (PR) differed importantly between countries with Italy showing the lowest participation rate (33%) and Poland the highest participation rate (95%) (Table 1). High participation rates were also reached in the Netherlands (78%), Sweden (73%), Denmark (71%), France (69%) and Spain (77 and 68%), while they were lower in Belgium (50%), Czech Republic (54%), Greece (48%) and Cyprus (52%).

Within the EXPOCHI project, all the food consumption databases containing the individual consumption data were harmonised to assure a uniform approach for the exposure assessments. All food items present in the different databases were categorised in food categories according to a pre-described system [23-25], in order to link the food items present in the different databases with the occurrence data in a harmonised way. EXPOCHI partners were shown how to categorise their food items according to this standardised system during a two-day food categorisation training course. These categorisation variables were included in the final food consumption databases submitted to EFSA. Furthermore, food codes and descriptors were provided and the consumption level (quantity) per food item (ingredient) summed up per day. Also, particular individual non-dietary information collected within the food consumption surveys was provided. The variables that were mandatory for all cases/subjects in the survey were: gender, age and bodyweight. We added the variable total energy intake per individual as an indicator of reporting completeness. In a few countries some missing values occurred for bodyweight. These values were replaced by the median of the children belonging to the same gender and age group.

All partners sent their food consumption database themselves to EFSA. The databases were not exchanged between partners. However, to standardise the format (variable names, labels and value names, labels and variable format) of these different databases, the coordinator provided a standard format for the food consumption databases. This standard format did not only facilitate the compilation of the different databases provided by the different partners, but also allowed the consortium to share syntaxes/scripts to be used in the exposure assessment studies.

**Figure 2:**
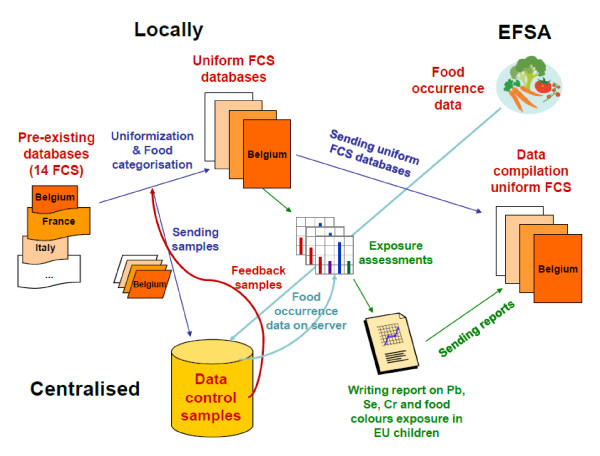
**Data flow, exposure assessments and reporting in EXPOCHI project**. FCS: food consumption survey).

In order to guarantee the uniformity level of the data submitted in a decentralised way to EFSA, samples of the databases were checked by the coordinator prior to submission (see Figure 2).

### 2. Exposure assessment in 1-14-year-old children to lead, chromium, selenium and food colours

The food consumption databases prepared/standardised during the first months of the EXPOCHI project (as described above) were used to assess the dietary exposure to lead, chromium, selenium and food colours [26-29]. These compounds demanded the performance of long-term exposure calculations since intake levels of these chemicals result in adverse effects that develop over a longer period of time. The input data required and the statistical methods used for the exposure assessments are described below.

#### Input data required

##### Food consumption data

The difficulty with long-term exposure assessments or usual dietary intake assessments, is that it is often not practical to collect food consumption data over long periods of time. Generally, food surveys are restricted to 2 up to 7 days per respondent (see Table 1 for details about the number of days collected within the different studies included in the EXPOCHI project). To perform long-term exposure assessments using these data, statistical models are needed to estimate usual dietary intakes/exposures. Models that separate the variation within persons from the variation between persons have proven to be very useful for this [30]. To use these models, at least two days per individual are required. Note that for Poland and Cyprus no long-term intake assessments could be performed up to the age of 10, as their food consumption database included only one 24 hr recall day per individual. Nevertheless, the food consumption data of Poland and Cyprus can be useful for EFSA for acute exposure assessments in the future and as Cyprus has data available for children from 11 to 14 years old, including at least two non-consecutive days, additional exposure assessments were reported for this older age group. Also the databases of the Czech Republic and both Spanish studies included food consumption data of children aged 11 to 14 years, and were addressed in the exposure assessments to chromium, lead and selenium. For food colours the interest was limited to children aged 1-10 years.

To link consumption data to occurrence data of lead, chromium and selenium, foods were categorised according to the food categorisation system used in the SCOOP Task report Task 3.2.11 [31]. In this SCOOP report 16 main food categories are defined (see Table 2). For the linking of food consumption data to concentration levels of food colours, the food categorisation was based on the one specified in the Council Directive 94/36/EC [32]. This food colour classification system was discussed with and tuned to the Flavourings, Additives and food Contact materials Exposure Task (FACET) project http://www.ucd.ie/facet/.

**Table 2:** Food categorisation system SCOOP 2004 [31]

MG	Main group
1	Dairy products and analogues (incl. dairy-based ice), excluding products of food category 2 (e.g. butter)

2	Fats, oils and fat emulsions (incl. imitation milks)

3	Edible ice (excl. dairy-based ice)

4	Fruits and vegetables (including mushrooms and fungi, roots and tubers, pulses and legumes, and aloe vera), seaweeds, and nuts and seeds

5	Confectionery

6	Cereals and cereal products (cereal grains, tubers, roots, pulses and legumes) (no bakery wares)

7	Bakery wares

8	Meat, meat products, poultry and game

9	Fish, fish products, molluscs, cephalopods, crustaceans and echinoderms (MCCE)

10	Eggs and egg products

11	Sweeteners (incl. honey)

12	Salt, spices, soups, sauces, salads and protein products

13	Foodstuff intended for particular nutritional uses

14	Beverages (excl. dairy products, imitation milks)

15	Ready-to-eat savouries

16	Composite foods

Recipes and composite foods were handled via standardised procedures. In food consumption databases, the composite foods/composed dishes/recipes can be available and indicated on different levels:

• as a recipe only (on recipe level only)

• disaggregated into its ingredients (on ingredient level only)

• both, as recipe and disaggregated into its ingredients (on recipe and ingredient level (in the same file))

If the food consumption data included information on both the recipe **and **the ingredient level, the partners were asked to add two variables that could distinguish between the ingredients and the composed dishes (see example in Table 3.).

**Table 3:** Example of the 2 variables added to distinguish between recipes and ingredients

recipe	ingred	food items in database
204	22	macaroni, cheese sauce and ham prepared

204	21	ham, cooked

204	21	sauce (milk-based)

204	21	macaroni, boiled without salt

recipe code: unique recipe code (a unique code for each different recipe of composed dish). ingred code: 22 was used to identify the recipe in the "ingred" variable; 21 was used to identify an ingredient of the recipe in the "ingred" variable.

The occurrence data of lead, chromium and selenium that we received from EFSA mainly concerned the ingredient level. However, some recipes (like pizza) were also included. If the occurrence data were available on the recipe level, the partners were recommended to do the linking on recipe level (selecting code 22 in the ingred variable). However, if no recipe occurrence data were available, partners had to split the recipe into ingredients to perform the linking on ingredient level (selecting code 21 in the ingred variable).

A more in-depth description and discussion of the food categorisation systems used within the EXPOCHI project is published in another manuscript [25]. In addition the food categorisation manuals that were used within EXPOCHI can be freely provided on request.

As mentioned before, within the EXPOCHI consortium only children from 1-14 years old were considered for analysis. However, it is noteworthy that the age ranges available in the different databases varied considerably (Table 1).

##### Occurrence data

EFSA provided the EXPOCHI consortium with the occurrence data of the substances under study in food and beverages (categorised in different food groups) in order to carry out the dietary exposure assessment studies. In the case of lead, chromium and selenium EFSA had launched Calls for data in order to collect information about their presence and occurrence in foods and beverages. These Calls are public requests to Member States for the provision of data they collect, mainly within monitoring activities, on the presence and occurrence of hazardous chemicals.

In the case of food colours, refined usage levels were requested from the industry. In addition, exposure was also assessed by assuming the presence of food colours at the Maximum Permitted Levels (MPLs) specified in the Directive of the European Parliament (1994) [32].

More information on the occurrence data is provided in the reports that have been published on the long-term exposure to the four substances [26-29].

##### Health-based limit values which characterise the hazard of the active substances

In quantitative risk assessments, the estimated dietary exposure is compared with a relevant health-based limit value (HBLV), like the acceptable daily intake (ADI) upper level (UL) or the tolerable daily intake (TDI) for long-term risk assessments. These HBLVs were obtained from the limit values as set and notified by Member States or based on internationally agreed values [33-35].

#### Statistical modelling

The focus of this study was the performance of long-term dietary exposure calculations by means of statistical models. These models allow the calculation of this type of dietary exposure using only the limited number of days (at least 2 days) usually present in the food consumption surveys, by removing the within-person variation from the total variation. By doing this, only the between-person variation is present, which is relevant to assess the long-term exposure. Several models have been developed based on this principle: including one developed by Slob [36] and another one developed by Nusser and co-workers at Iowa State University (ISU-method) [37-39]. These models are not implemented in statistical software packages such as SPSS or SAS. However, both models (Slob & Nusser) have been programmed in the Monte Carlo Risk Assessment (MCRA) software, developed by RIKILT - Institute of Food Safety (The Netherlands) [40]. In EXPOCHI we used the Slob method only. This modelling approach results in a distribution of long-term (or usual) dietary exposure levels in the relevant population group. For the estimation of the long-term exposure, daily consumption patterns (e.g., 2, 558 measurements, 2 days × 1, 279 Dutch children) were multiplied by the food group-specific Lower Bound (LB) and Upper Bound (UB) mean metal concentrations, and added to foods consumed per day per individual. In this way, the whole diet was addressed when assessing the exposure to the specific metal/element. The estimated exposures were adjusted for the individuals' body weight.

A distribution of daily metal/element exposures, calculated as described above using mean concentrations per food group and adding the exposure to foods, includes both the variation between individuals and between days within individuals. However, to assess the long-term intake within a population only the former type of variation is of interest, since in the long run the intake between different days of one individual will level out. Therefore, to calculate a long-term dietary exposure distribution, the distribution of daily exposures should first be corrected for the within-person (between days) variation using statistical models. In EXPOCHI, the betabinomial-normal (BBN) model [36,41] was used for this. Shortly, the BBN model refers to a betabinomial (BB) distribution used for modelling exposure frequencies and a normal (N) distribution used for modelling positive exposures on a suitably chosen transformed scale. Exposure frequency modelling concerns the number of days for which an exposure is recorded. This number of days is assumed to have a binomial distribution, with a binomial index equal to the total observed number of days, and as parameter the probability of exposure, which may itself vary between individual persons. More details about this betabinomial-normal (BBN) model were published previously by De Boer and Van der Voet [36,41].

However, in order to use these models to assess the long-term exposure certain conditions need to be fulfilled (e.g. related to the distribution of the intakes: normal distributions are required). If these conditions were not met, a deterministic approach was used to assess the exposure. The deterministic approach has also been implemented in the MCRA-software. For this, the following formula was used to estimate the intake of the component per child per kilogram of body weight: Intake _per person _= ∑(Xi * Yi)/BW

X_i _= amount (g) of food group i consumed per respondent per day (mean of different days)

Y_i _= mean occurrence of the component in the food group i (g/10^z^g)

BW = body weight of child (kg)

∑ = sum of all food groups

In the EXPOCHI project all partners used the MCRA software via the internet to calculate the long-term exposure using their own food consumption data, following standardised procedures. All partners attended a two-day training programme to assist them in the use of the MCRA software [41].

### 3. Timeline of the project

Prior to the project and during the first month of the project, information about the different national/regional survey data that were included in the EXPOCHI project were collected (sampling procedures, dietary intake assessment method used, number of days included, age group, etc.). During the same period, a database template was developed by the coordinator to allow for a standard approach in all countries to classify national foods. In the second month, a kick-off meeting, combined with a training course on the food categorisation to be used for the exposure assessments to lead, chromium selenium, was organised by the coordinator. In this training, important methodological issues were discussed, such as the age groups to include for the different countries and how to use the German cohort food consumption data. Regarding the ages to be included it was agreed to only include children younger than the age of 15 in the EXPOCHI project. Adolescents of 15 years or older were insufficiently available in the different national databases and already sufficiently covered by most of the national food consumption surveys among adults (see EFCOSUM recommendations [42]). Furthermore, Poland was not included in the EXPOCHI reports describing the exposure results of the four compounds [26-29], since the survey only included one 24 hr recall day. During the third month, all partners applied the food categorisation system to their food consumption databases. In the fourth month, when all food categorisation was finished, a training course on the use of the MCRA software to perform the long-term exposure assessments was given by RIKILT - Institute of Food Safety. EFSA also participated in this workshop, allowing in-depth discussions about remaining methodological issues, such as the categorisation of the occurrence data and the timeline for providing the consortium with this data. During this workshop, the deadlines for the deliverables were revised based upon the timeline for the occurrence data provided by EFSA. In this workshop, EFSA also requested the consortium to run an additional (more in-depth) analysis for the food colour lycopene to allow inclusion of these results in the EFSA opinion on lycopene [27,43].

During the following two months, the exposure assessments for lead were executed and compiled in a report during month seven of the project. In month eight, a food categorisation system was developed for the food colour exposure assessments and was applied to the food consumption databases in month 9 and 10. In month 10, a third EXPOCHI workshop was organised to discuss the results obtained so far and the procedures that were applied. During this third workshop important quality control procedures were discussed. This discussion resulted in the execution of additional quality checks regarding the four different components under study (lead, chromium, selenium and food colours). For these quality checks, a tiered approach was applied:

#### Tier 1. Logical 'control' Comparison of the average energy intake calculated for individual ages (years) with the estimated energy expenditures

Justification: the group average for energy intake calculated from national food consumption data should be in a range roughly comparable with energy expenditure as calculated by Basal metabolic Rate multiplied by the Physical activity level (BMR*PAL) [44]. Simply put, energy intake should be more or less comparable between countries. If not, other comparisons of EXPOCHI results between countries can be problematic due to 'data complexity/reliability'. This comparison was performed for all countries and reported in the selenium exposure report [29], and did not result in adjustment.

#### Tier 2. 'Control' of the number of foods classified for a particular food category between food surveys

Justification: this comparison may disclose the approach of food classification in countries -- real or biased classification done by individual experts. This cross-check was performed for the two Spanish food consumption databases and based upon this control check, some extra reclassifications were done.

#### Tier 3. 'Control' of principal contributors to exposure

Justification: very 'large and small contributors to exposure' were checked for their reliability by checking the food items involved in food groups contributing largely to the exposure. If unrealistic results were found, the calculations were redone. If the contribution of the foods was similar over the different countries this was seen as an indication of reliable results.

All other exposure assessments (chromium, selenium and food colours) were performed in months 11 and 12. Simultaneously, all the reports on the exposure assessment studies were drafted during these two months.

A schematic overview of the data flow, the exposure assessments and the reporting of the exposure results is given in Figure 2. The EXPOCHI-Manual on Food Categorisation for the Lead, Chromium and Selenium exposure assessments [23] and for food colour assessments [24] were developed centrally by the coordinator and distributed to the different partners who categorised the food items from their survey according to the harmonized procedures explained in the manual. A sample of the categorised food consumption database was sent to the coordinator who checked the food categorisation procedure performed by the partners. The coordinator sent its comments/corrections to the partners so that they could correct their classification procedure if necessary. The occurrence data were provided by EFSA and were checked for consistency and extreme values by the coordinator (some corrections were made based upon these checks and after having consulted EFSA). The occurrence data were uploaded on the MCRA server and all partners individually uploaded their food consumption data on the MCRA server. When the occurrence data and food consumption data were uploaded, the partners could start the exposure assessment procedures using the MCRA software. RIKILT functioned as helpdesk for all exposure assessment analyses. The results of the exposure assessments were sent to the coordinator and to RIKILT who were responsible for the compilation of the results in four exposure assessment reports (the EXPOCHI deliverables). At the end of the project, all partners had to send their food consumption database that was classified according to the EXPOCHI manuals to EFSA (the final EXPOCHI deliverable).

## Discussion

EXPOCHI includes 14 national/regional food consumption databases available for children, using different dietary assessment methodologies. These databases were used for long-term exposure assessment of lead, chromium, selenium and food colours in children, using occurrence data provided by EFSA. These reports have been published on the EFSA website [26-29]. All foods were categorised via a standardised EXPOCHI food categorisation system and analysed via uniform procedures, using the MCRA software [36]. The linkage of the food consumption data to the occurrence data was also performed in a harmonised way.

### Methodological considerations

Important strengths of this large-scale exposure assessment study among European children are the large amount of food consumption data, from surveys undertaken across Europe, the application of a standardised food categorisation system and the uniform way in which the exposure assessments via MCRA were performed.

This is the first time that dietary exposure assessments for children are performed on such a large scale (covering different regions in Europe). Also the fact that the food items of 14 different European consumption databases are now categorised in a uniform way creates possibilities to use these categorised databases for other goals beyond the EXPOCHI project.

### The collaborative approach preparing and discussing the results to enhance the critical interpretation of such sensitive figures is another strength of this study

However, this EXPOCHI project was also prone to certain limitations, which are discussed in more depth below.

#### The data used

The different **food consumption databases **used within the EXPOCHI project were based on different survey designs, using different dietary assessment methods. They consisted of the data available at national/regional level for food safety purposes and are representative of national/regional dietary patterns. The level of detail in which the food consumption was recorded differed however per country, and consequently affected the accuracy of the food categorisations and exposure assessments of the different partners. Due to differences between the individual surveys regarding e.g. sampling design, the methodology used to assess the food consumption levels and age groups addressed, it was not feasible to use the outcomes of the exposure assessments to make comparisons between countries or regions. Also the fact that no sampling weights could be used in the MCRA software further limited the comparability of the study outcomes between countries (some of the surveys require weighing factors to guarantee the representativity of the results).

Also the **occurrence data **used in the project had limitations. First the food occurrence data that EFSA received from the EU countries covered a wide range of different (national) foods, including both raw and processed foods. To use these data for exposure calculations, EFSA aggregated them into major food groups, resulting in rather rough intake estimations. How well these 'European' concentration data represent levels to which children in Europe are exposed, will depend on many things. In view of the international trade developments with foods traversing the whole of Europe to reach the consumers, a European concentration database is an option that may come close to what a European consumer may encounter in real life. However, for this the data in the European database should represent the levels to which children may be exposed, covering many countries that are evenly represented in the database. Furthermore, despite international trade developments contamination levels may still differ greatly per country due to e.g. environmental differences. In those cases, the use of a European contamination database may result in wrong conclusions. There are other sources of uncertainty related to the use of occurrence data at a European level which will be addressed in more detail in the papers dealing with the exposure to the compounds studied in this project.

### Exposure assessments

Not all partners within the project were able to conduct all four exposure assessments. Poland could not run the exposure assessments since they only had one single 24 hr recall day, while at least two independent days are required to assess usual intake. The food consumption databases prepared for the assessments, including the food categorisation used to link the food consumption data to the occurrence data of the four chemicals addressed, were supplied to EFSA. Consequently, EFSA will be able to rerun the analyses including all the national/regional databases and will have the possibilities to run other scenarios and to calculate dietary exposure of other compounds categorised in a similar way.

It should be noted that exposure assessments across Europe using harmonised food consumption databases and common chemical occurrence databases, as well as a common exposure assessment method enabled us to compare the exposures between countries or European regions (with methodological reservations) and showed us the methodological problems such comparisons entail. More details on these problems have been addressed in the reports discussing the exposure to the individual chemicals [26-29].

#### Sampling and representativeness

Due to the fact that data from large surveys undertaken in thirteen different countries were available, a good geographical spread over Europe was guaranteed. (Figure 1).

Although the different national/regional food consumption surveys included in the EXPOCHI project were representative for a certain age group within that particular country/region, we cannot say that the compiled sample of children included in the EXPOCHI project, was representative of all European children aged 1-14 years. First of all, the youngest age group (children 1 year old) are underrepresented compared to the older children (as presented in Table 1, only few countries included data on young children). Furthermore, the methodology used to conduct the surveys was not harmonised and aimed at guaranteeing results representative at a European level.

### Guarantee for quality level of scientific output

As shown in Figure 2, special attention in the EXPOCHI project was given to the different quality procedures and checks. The coordinator was responsible for the quality checks of the different food consumption surveys used. To check the categorisation of the national foods in the different food categories used to assess the exposure to the selected compounds, all partners sent a sample (including at least 25% of all the food items included in their database (most partners provided their whole database)) to the coordinator so that the categorisation procedures could be checked for all countries. Also the occurrence data obtained from EFSA were critically checked by the coordinator (in collaboration with RIKILT). Doubtful or implausible values were highlighted and were double-checked by EFSA. Finally, the results of the exposure assessments were checked by all partners and were openly discussed during the 3^rd ^workshop. This in-depth discussion focused mainly on the limitations of food consumption and concentration data used and the results obtained, which allowed a critical appraisal of our data and results. As discussed before, a tiered approach was applied in analysing our food consumption data in relation to the exposure assessment results.

In addition, an excellent level of scientific output for this project was guaranteed by the collaboration of different partners across Europe having a long-standing experience in collecting individual food consumption data as well as in exposure assessment studies. Moreover, most of the partners collaborate in large European projects related to food consumption and exposure assessment. The pre-existing knowhow and expertise of the different partners together allowed in-depth and fruitful discussion of the different methodological issues that needed to be handled in such a challenging project as EXPOCHI.

Furthermore, for the food colours intake study, particular attention was given to other on-going works on food additive intake assessments, namely the DG Sanco 'ad hoc working group on food additives intake' and the food additive work package of the FACET project. Some EXPOCHI partners participate in the FACET project (AFSSA and INRAN).

### Future possibilities of EXPOCHI databases

In the future, EFSA plans to use the uniform food consumption databases derived from the EXPOCHI project within EFSA opinions. They have already done so regarding an EFSA opinion on arsenic, published in February 2010. All partners get the chance to give input or comment on those opinions in which their food consumption data is used prior to publication, to avoid errors due to misinterpretations of the EXPOCHI food consumption data.

To conclude, the problems and solutions handled within this project will contribute to the optimisation of future harmonised exposure assessment studies among children in Europe. One of the most important conclusions from our survey is that harmonisation of the dietary intake assessment methods and sampling designs used to collect food consumption are crucial to guarantee an optimal comparability between countries. This lack of standardisation during data collection was an important limitation in our EXPOCHI project. In addition, the broad (aggregated) food categories that were used for the occurrence data limited the precision of the exposure assessments which was dealt with by projecting worst-case scenarios'.

## List of abbreviations used

ADI: acceptable daily intake; BBN: Betabinominal-normal; Bw: Body weight; DIPP study: The Finnish Type I Diabetes Prediction and Prevention Study; EFSA: European Food Safety Authority; enKid Study: Food patterns of Spanish schoolchildren and adolescents; EXPOCHI: Individual food consumption data and exposure assessment studies for children; FACET: Flavourings; Additives and food Contact materials Exposure Task; HBLV: health-based limit value; INCA 2: Second French Individual National Food Consumption Survey; ISU: Iowa State University; JECFA: Joint; FAO/WHO Expert Committee on Food Additives; LB: Lower bound; MCRA: Monte Carlo Risk Assessment; MPLs: Maximum Permitted Levels; OIM: Observed individual means; SCOOP: Scientific Cooperation; SISP04: Czech Republic - Individual food consumption study; STRIP study: Special Turku Coronary Risk Factor Intervention Project; TDI: tolerable daily intake and UL: upper level.

## Competing interests

The authors declare that they have no competing interests.

## Authors' contributions

All authors participated in the design of the study, the exposure assessments and drafting of the manuscript. The coordination of the study was executed by Ghent University in close collaboration with RIKILT - Institute of Food Safety. All authors read and approved the final manuscript.
